# Insulitis in human diabetes: a histological evaluation of donor pancreases

**DOI:** 10.1007/s00125-016-4140-z

**Published:** 2016-10-28

**Authors:** Marcus Lundberg, Peter Seiron, Sofie Ingvast, Olle Korsgren, Oskar Skog

**Affiliations:** Department of Immunology, Genetics and Pathology, The Rudbeck Laboratory C11, Clinical Immunology, Uppsala University, Dag Hammarskjölds väg 20, 751 85 Uppsala, Sweden

**Keywords:** HLA, Inflammation, Insulin secretion, Insulitis, Islets, Macrophages, T cells, Type 1 diabetes, Type 2 diabetes

## Abstract

**Aims/hypothesis:**

According to the consensus criteria developed for type 1 diabetes, an individual can be diagnosed with insulitis when ≥ 15 CD45^+^ cells are found within the parenchyma or in the islet–exocrine interface in ≥ 3 islets. The aim of this study was to determine the frequency of individuals with type 2 diabetes fulfilling these criteria with reference to non-diabetic and type 1 diabetic individuals.

**Methods:**

Insulitis was determined by examining CD45^+^ cells in the pancreases of 50, 13 and 44 organ donors with type 2 diabetes, type 1 diabetes and no diabetes, respectively. CD3^+^ cells (T cells) infiltrating the islets were evaluated in insulitic donors. In insulitic donors with type 2 diabetes, the pancreases were characterised according to the presence of CD68 (macrophages), myeloperoxidase (MPO; neutrophils), CD3, CD20 (B cells) and HLA class I hyperstained islets. In all type 2 diabetic donors, potential correlations of insulitis with dynamic glucose-stimulated insulin secretion in vitro or age, BMI, HbA_1c_ or autoantibody positivity were examined.

**Results:**

Overall, 28% of the type 2 diabetic donors fulfilled the consensus criteria for insulitis developed for type 1 diabetes. Of the type 1 diabetic donors, 31% fulfilled the criteria. None of the non-diabetic donors met the criteria. Only type 1 diabetic donors had ≥ 15 CD3^+^ cells in ≥ 3 islets. Type 2 diabetic donors with insulitis also had a substantial number of CD45^+^ cells in the exocrine parenchyma. Macrophages constituted the largest fraction of CD45^+^ cells, followed by neutrophils and T cells. Of type 2 diabetic pancreases with insulitis, 36% contained islets that hyperstained for HLA class I. Isolated islets from type 2 diabetic donors secreted less insulin than controls, although with preserved dynamics. Insulitis in the type 2 diabetic donors did not correlate with glucose-stimulated insulin secretion, the presence of autoantibodies, BMI or HbA_1c_.

**Conclusions/interpretation:**

The current definition of insulitis cannot be used to distinguish pancreases retrieved from individuals with type 1 diabetes from those with type 2 diabetes. On the basis of our findings, we propose a revised definition of insulitis, with a positive diagnosis when ≥ 15 CD3^+^ cells, not CD45^+^ cells, are found in ≥ 3 islets.

**Electronic supplementary material:**

The online version of this article (doi:10.1007/s00125-016-4140-z) contains peer-reviewed but unedited supplementary material, which is available to authorised users.

## Introduction

In type 1 diabetes, leucocyte infiltration of islets (insulitis) has a presumed central role in beta cell destruction and has been regarded evidence for an autoimmune aetiology of the disease [1, 2]. Different criteria have been used to define insulitis [2], making it difficult to compare and analyse results from different studies. A consensus opinion was recently reached on the criteria for insulitis: a patient is diagnosed with insulitis when a lesion is established in a minimum of three islets with a threshold level of ≥ 15 CD45^+^ cells within the islet parenchyma or in the islet–exocrine interface [1]. CD45, also known as protein tyrosine phosphatase receptor type C, is an enzyme specifically expressed on haematopoietic cells. Macrophages, neutrophils, T cells and B cells have been reported to be part of the insulitic infiltrate in type 1 diabetes [2–5]. At the time when the consensus definition was established, seven was the highest number of CD45^+^ cells found in islets from 61 non-diabetic control pancreases examined in the Network for Pancreatic Organ Donors with Diabetes (nPOD) [1, 6], and thus the consensus definition adequately separated non-diabetic controls from individuals with type 1 diabetes.

Inflammation of the pancreas has been suggested to be part of the aetiopathology of type 2 diabetes [7]. Supporting this theory, an elevated number of macrophages have been detected in islets of type 2 diabetes patients in conjunction with increased levels of cytokines and chemokines [8]; CD8^+^ cells and macrophages have also been found to be elevated in the exocrine compartment in type 2 as well as type 1 diabetes [9, 10]. Furthermore, IL-1 receptor antagonist treatment of type 2 diabetes patients reduces HbA_1c_ and enhances C-peptide levels, but does not alter insulin resistance [11], suggesting a direct role of inflammation in type 2 diabetes islet dysfunction. Collectively, these observations suggest that inflammation of the pancreas plays a prominent pathogenic role in type 2 diabetes.

In this study we determined the frequency of individuals diagnosed with type 2 diabetes fulfilling the definition of insulitis according to the consensus criteria developed for type 1 diabetes. Further, in pancreases with insulitic lesions, the number of macrophages, neutrophils, T cells and B cells was determined. In addition, other morphological hallmarks for type 1 diabetes were evaluated in the type 2 diabetic pancreases, such as islets without beta cells [2, 12, 13] or the presence of HLA class I hyperstaining in the islets [14, 15]. Finally, potential correlations between the presence of insulitic lesions and glucose-stimulated insulin secretion in vitro or clinical characteristics such as age, BMI, HbA_1c_ and autoantibody positivity of the donor were examined.

## Methods

### Human pancreatic specimens

Biopsies from the pancreases of 50 donors with type 2 diabetes based on established clinical diagnosis or on HbA_1c_ > 6.5% (48 mmol/mol) [16], 13 donors with type 1 diabetes and 44 previously healthy organ donors with HbA_1c_ < 6.0% (42 mmol/mol) were included in the study. The medical records of the donors were not made available in order to protect the integrity of the deceased person, but most donors with type 2 diabetes had been diagnosed for several years. Of the type 1 diabetic donors, two died at onset (described previously in Korsgren et al [5]) and 11 had been diagnosed for > 1 year. Consent for organ donation (for clinical transplantation and for use in research) was obtained verbally from the deceased’s next of kin by the attending physician and was documented in the medical records of the deceased in accordance with Swedish law and as approved by the Regional Ethics Committee (Dnr 2015/444). The type 2 diabetes donors were, on average, aged 63 years (range 44–85) with BMI 28 kg/m^2^ (range 20–42). The non-diabetic donors were matched for age (59 years [42–72]), sex and BMI (27 kg/m^2^ [21–46]) to the type 2 diabetes donors. The donors with type 1 diabetes had a mean age of 39 years (16–68). The pancreatic tails of the type 2 diabetes donors were used for islet isolation, and tissue samples were taken from the pancreas, fixed in paraformaldehyde and embedded in paraffin. One to two tissue samples from different parts of the type 2 diabetes pancreases, one tissue sample from the head of the non-diabetic pancreas collected during the process of clinical islet isolation, and a minimum of two samples from different parts of the type 1 diabetes pancreases were included in the study.

### Immunohistochemical staining

Consecutive sections (6 μm) from each tissue sample block were processed and labelled using a standard technique. All antigens were unmasked by heat-induced antigen retrieval using buffered sodium citrate or EDTA according to the manufacturer’s instructions (Dako, Glostrup, Denmark). Antibodies against synaptophysin and CD45 (electronic supplementary material [ESM] Table 1) were applied. In donors with insulitis (≥15 CD45^+^ cells in ≥ 3 islets), sections consecutive to the section stained for synaptophysin/CD45 were stained for CD68 (macrophages), myeloperoxidase (MPO; granulocytes), CD3 (T cells), CD20 (B cells), insulin and glucagon, HLA class I ABC and active caspase 3. Human spleen sections were used as a positive control for all antibodies except caspase 3 and HLA, for which human tonsil sections were used. Negative controls had the primary antibody replaced by buffer. Immunohistochemical reactions were performed on paraffin tissue sections using a manual method or an automated immunohistochemical stainer (Autostainer Link 48, Dako, Glostrup, Denmark). All bound primary antibodies except caspase-3 were visualised using Dako EnVision and diaminobenzidine (DAB)-based substrate or double stained using EnVision G/2 Doublestain System, Rabbit/Mouse (DAB^+^/Permanent Red). Caspase-3 antibodies were visualised using MACH1 Universal HRP-Polymer Detection kit (Biocare Medical, Concord, CA, USA). Sections were counterstained with haematoxylin and analysed using a light microscope (Leica DM 2000 LED, Wetzlar, Germany).

### Analysis

Donors were diagnosed with insulitis when ≥ 15 CD45^+^ cells within the islet parenchyma and/or in the islet–exocrine interface were found in ≥ 3 islets. CD45^+^ cells adjacent to an islet or in direct contact with other CD45^+^ cells within the peri-insulitic cap were counted as being part of the insulitic lesion. A total of 250 (SD = 189), 118 (SD = 54) and 48 (SD = 36) islets per donor were analysed for insulitis in type 2 diabetic, type 1 diabetic and non-diabetic donors, respectively. At the time of analysis, the examiner knew only the disease status of the donor. In donors diagnosed with insulitis, the number of CD3^+^ cells associated with islets were counted in the same manner as CD45^+^ cells and the number of cells stained for active caspase-3 were counted in islets. Insulin and glucagon content in islets was estimated and insulin-deficient islets were noted.

**Fig. 1:**
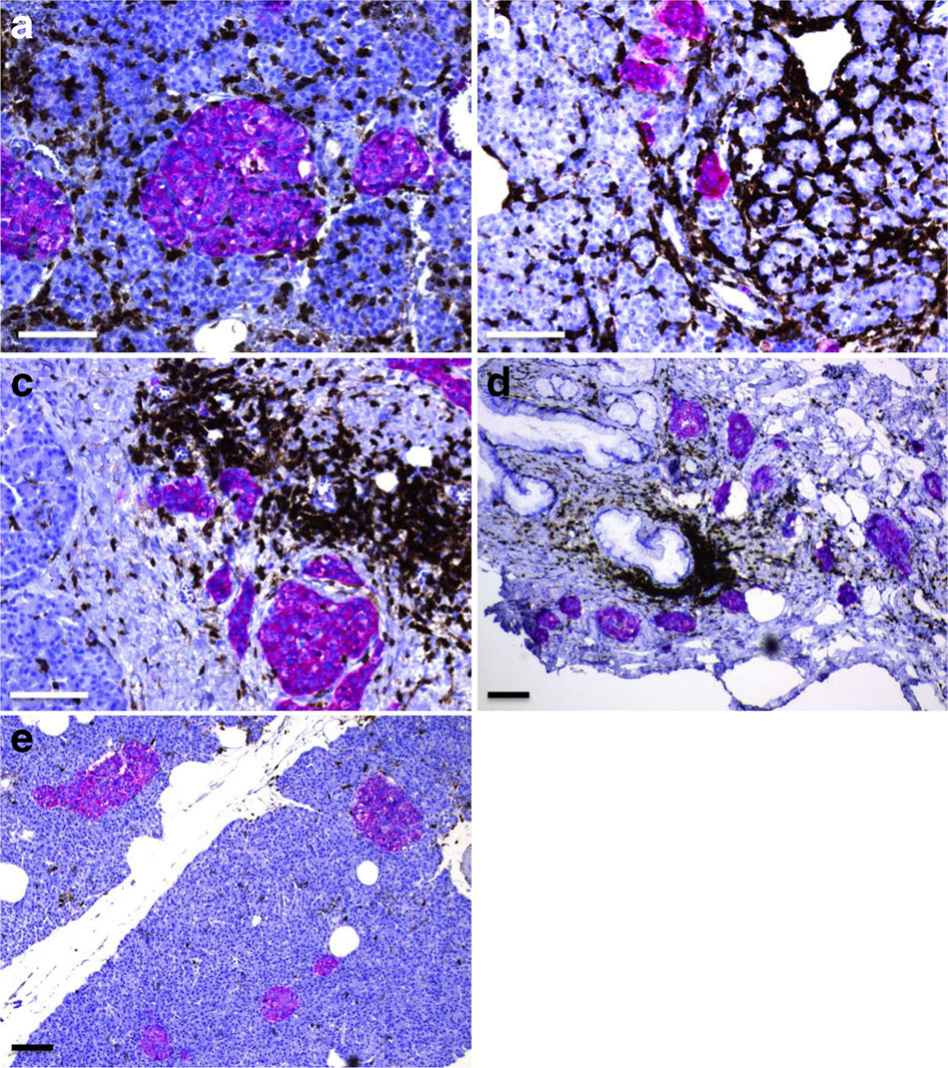
Insulitis in type 2 diabetes. Pancreatic sections were stained for CD45 (brown) and synaptophysin (red). Representative pictures are shown of areas in the pancreas with insulitic islets and a high density of CD45^+^ cells (**a**, **b**) and of fibrotic areas with islets and a rich amount of CD45^+^ cells (**c**, **d**). Some type 2 diabetic pancreases contained few CD45^+^ cells, as illustrated in (**e**). Parts (**a**, **b**), (**c**, **d**), and (**e**) are from three different donors with type 2 diabetes. Scale bars, 100 μm

**Fig. 2:**
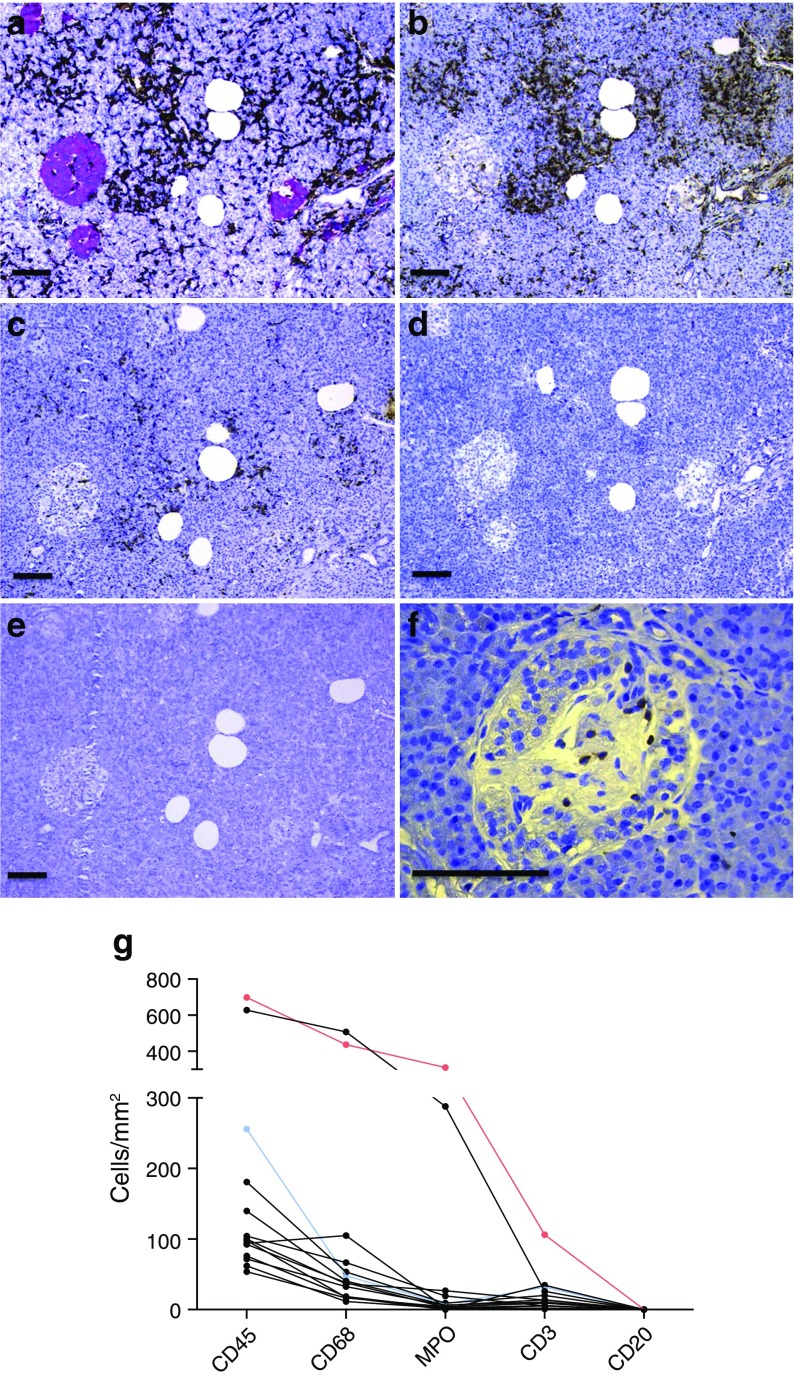
CD68^+^ cells were the most prominent immune cell type in insulitic type 2 diabetic pancreases. The densities of CD45^+^ (**a**), CD68^+^ (**b**), MPO^+^ (**c**), CD3^+^ (**d**) and CD20^+^ (**e**) cells were determined in consecutive sections in each type 2 diabetic donor diagnosed with insulitis. All images (**a**–**e**) are from one donor. Synaptophysin was stained red in (**a**) and immune cells were stained brown. The densities of immune cells were compared in 14 donors with type 2 diabetes (**g**). The red dots in (**g**) represent the donor from whom the samples used in (**a**–**e**) were taken, with relatively high numbers but typical distribution of immune cells. The blue dots represent a donor who had several islets with relatively many islet-associated CD3^+^ cells (7, 9 and 10 CD3^+^ cells in three different islets). One such islet is depicted in (**f**). Scale bars, 100 μm

In type 2 diabetic donors, the disposition and amount of connective tissue, acinar tissue, endocrine tissue and CD45^+^ immune cells were evaluated in all sections stained for CD45 and synaptophysin. The pancreatic tissue was graded according to the presence of CD45^+^ cells: (1) only a few CD45^+^ cells, scattered in the pancreatic tissue (example in Fig. 1e); (2) an intermediate number of CD45^+^ cells; and (3) intense CD45^+^ inflammation, with immune cells infiltrating the tissue in large numbers (examples in Fig. 1a–d). In insulitic type 2 diabetic donor sections, CD45^+^ cells were quantified by counting in a randomly selected area of approximately 1 mm^2^, and CD68^+^, MPO^+^, CD3^+^ and CD20^+^ cells were counted in the corresponding area in consecutive sections. A donor was diagnosed with HLA class I hyperstaining when one or more islets within a pancreas displayed intense staining for HLA class I ABC. Clinical data regarding age, BMI, HbA_1c_ and autoantibody positivity, collected by the Nordic Network for Islet Transplantation, were compared between non-insulitic and insulitic type 2 diabetes donors.

### Insulin secretion

Islets were isolated using a standard protocol [17]. One day after isolation, glucose-stimulated insulin secretion was assessed in a dynamic perifusion system, Suprafusion 1000 (BRANDEL, Gaithersburg, MD, USA). Twenty handpicked islets were perifused with low glucose (1.67 mmol/l) for 42 min, high glucose (20 mmol/l) for 48 min, and then low glucose again. Fractions were collected at different time points and secreted insulin was measured using insulin ELISA (Mercodia, Uppsala, Sweden).

### Statistical analysis

GraphPad Prism software (version 6.0 h) was used for statistical analysis. The Mann–Whitney *U* test was used to compare age, BMI and HbA_1c_ between non-insulitic and insulitic type 2 diabetic donors. A *p* value < 0.05 was considered statistically significant.

## Results

### Type 2 diabetic donors fulfilled the consensus criteria for insulitis

Using the consensus definition of insulitis from 2013 (≥3 islets with ≥ 15 CD45^+^ cells), 14 out of 50 (28%) type 2 diabetic donors fulfilled the criteria for insulitis. In these donors, the mean frequency of insulitic islets was 2.8% (SD = 2.3). CD45^+^ cells were also found scattered in the exocrine tissue (Fig. 1a, b), with no obvious association with islets. Insulitic islets occurred in seven of 36 pancreases containing a low density of CD45^+^ cells in the exocrine pancreas, six of 13 pancreases containing an intermediate density of CD45^+^ cells and one of one pancreas containing an intense density of CD45^+^ cells (Fig. 1a, b). Of 50 type 2 diabetic donors, 41 (82%) were diagnosed with insulitis using the old definition of insulitis (≥3 islets with ≥ 5 CD45^+^ cells) [2].

Of 50 donors with type 2 diabetes, 17 had fibrotic areas with islets (Fig. 1c, d). In 15 of these 17 cases, the fibrotic area was also rich in CD45^+^ cells, and consequently, some of the islets fulfilled the criteria for insulitis, i.e. six of these donors were diagnosed with insulitis. The intensity of inflammation appeared heterogeneous between different lobes within a type 2 diabetic pancreas, as well as between individuals (Fig. 1e). In the type 1 diabetic group, four of 13 donors (31%)—including both of those with acute-onset disease—were diagnosed with insulitis. The mean frequency of insulitic islets in these donors was 4.2% (SD = 1.8). None of the non-diabetic donors fulfilled the consensus definition criteria. Indeed, only one islet of a total of 2130 islets in non-diabetic donors had ≥ 15 CD45^+^ cells. Ten of 13 type 1 diabetic donors and ten of 44 non-diabetic donors fulfilled the old criteria for insulitis (≥3 islets with ≥ 5 CD45^+^ cells).

### Macrophages were the most frequent immune cell in the insulitic type 2 diabetic pancreases

In the 14 insulitic type 2 diabetic pancreases, on average 102 CD68^+^ cells were found per mm^2^. In addition, MPO and CD3^+^ cells were regularly found (49 and 20 cells/mm^2^, respectively) (Fig. 2a–f). CD20^+^ cells were found only occasionally. The highest number of CD3^+^ cells found within and/or at the periphery of an islet was 18; multiple islets were also found with 6–10 CD3^+^ cells (Fig. 2g), but there were no findings of ≥ 15 CD3^+^ cells in ≥ 3 islets. In comparison, both donors with acute-onset type 1 diabetes had ≥ 3 islets with ≥ 15 CD3^+^ cells (6/51 and 3/38 islets, respectively), whereas none of the two insulitic donors with long-standing type 1 diabetes fulfilled this criterion (0/128 and 2/54 islets with ≥ 15 CD3^+^ cells, respectively).

Activated caspase-3 was rarely found in insulitic donors, with a frequency of 1–2 positive cells per 100 islets and with no apparent difference between individuals with type 2 diabetes and long-standing type 1 diabetes. However, positive cells were found at about ten times higher frequency in the donors with acute-onset type 1 diabetes.

### Insulin-deficient islets were rare in insulitic type 2 diabetic pancreases

**Fig. 3:**
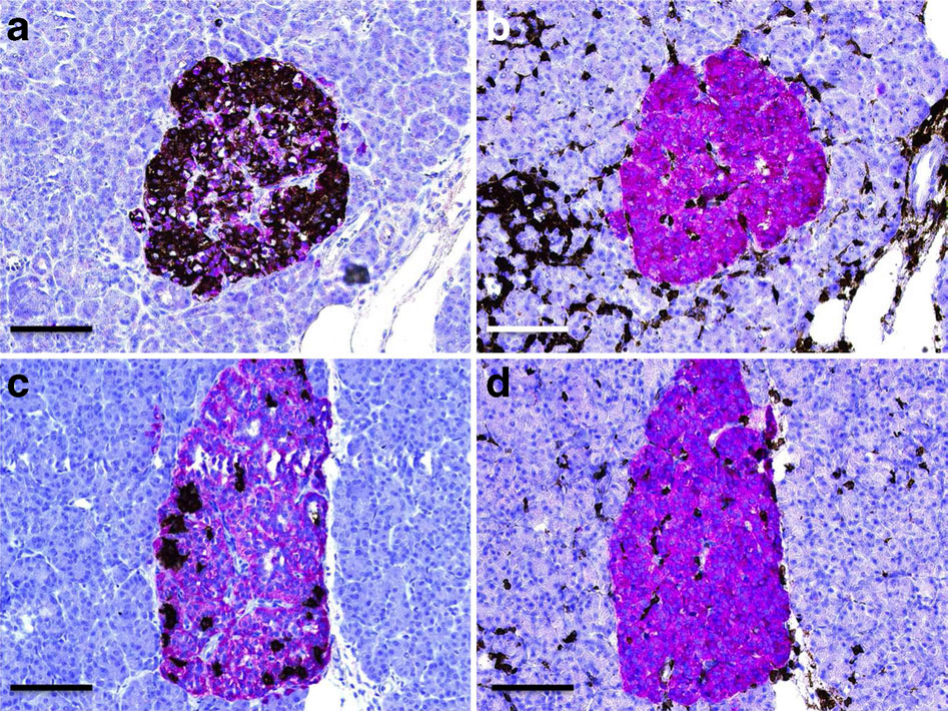
Insulitis affected islets regardless of insulin content. (**a**, **c**) Islets stained for insulin (brown) and glucagon (red). A representative islet with ‘normal’ distribution of insulin and glucagon (**a**) and an islet with few insulin-positive cells (**c**) are shown. (**b**, **d**) The same islets in consecutive sections stained for CD45 (brown) and synaptophysin (red). Parts (**a**, **b**) and (**c**, **d**) are from two different donors. Scale bars, 100 μm

Most islets in the type 2 diabetic individuals contained both insulin- and glucagon-positive cells (Fig. 3a, b). In total, fewer than five islets devoid of beta cells were found from all of the 2831 islets examined in insulitic type 2 diabetic pancreases. Insulitis was most commonly found in islets containing both insulin- and glucagon-positive cells, but could also be found in the rare islets consisting of only a few insulin-positive cells (Fig. 3c, d). In contrast, all the pancreases of type 1 diabetic donors contained insulin-deficient islets. Both donors with acute-onset type 1 diabetes and one donor with long-standing type 1 diabetes had numerous insulin-containing islets. These donors also fulfilled the criteria for insulitis according to the consensus definition. The remaining ten donors with long-standing type 1 diabetes had almost solely insulin-deficient islets, but a few scattered insulin-positive cells were found in two cases.

### HLA class I hyperstained islets occurred in insulitic type 2 diabetic donors

**Fig. 4:**
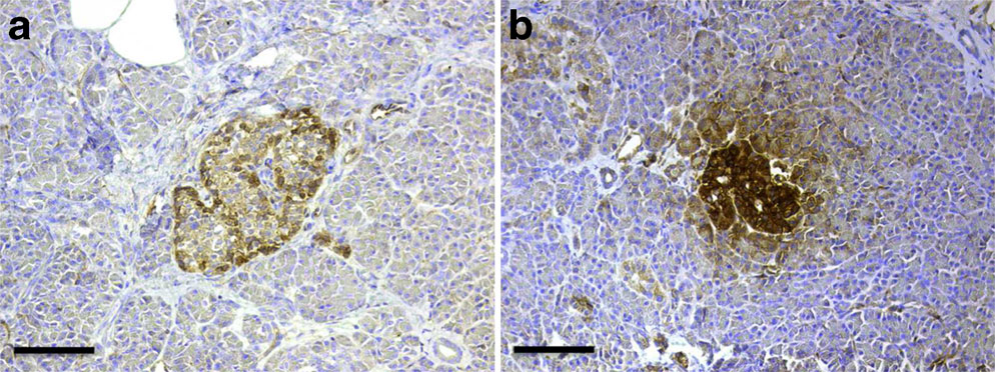
HLA class I hyperstained islets were found in type 2 diabetic pancreases. HLA class I (brown) in insulitic type 2 diabetic donors. Representative islets graded as non-hyperstained (**a**) and hyperstained (**b**) are shown. Parts (**a**) and (**b**) are from two different donors. Scale bars, 100 μm

Five of 14 (36%) of the insulitic type 2 diabetic pancreases contained at least one islet with intense staining for HLA class I (see Fig. 4 for representative pictures). This hyperstaining affected islets both with and without insulitis.

### Glucose-stimulated insulin secretion was similar in type 2 diabetic donors with and without insulitis

**Fig. 5:**
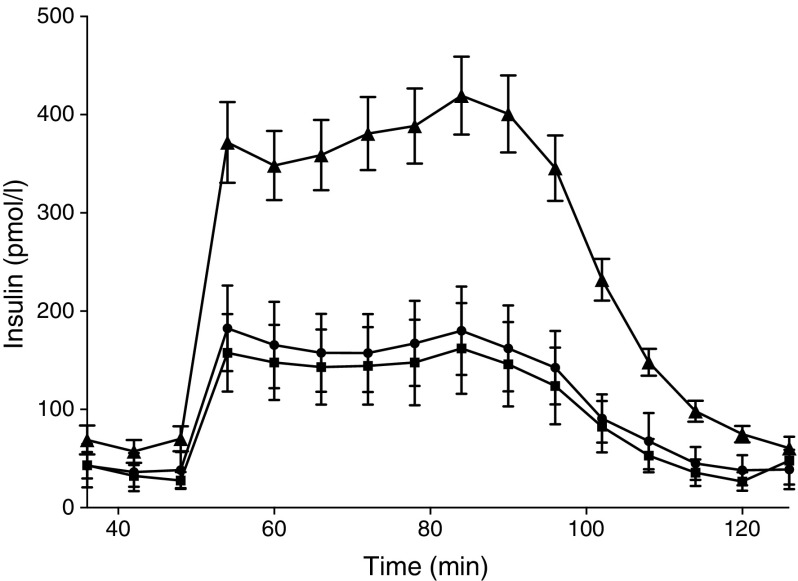
Glucose-stimulated insulin secretion in islets isolated from type 2 diabetic donors. Isolated islets were stimulated in a dynamic perifusion assay. Insulin secretion was compared between age-, BMI- and sex-matched non-diabetic controls (*n* = 38, triangles), and non-insulitic (*n* = 27, circles) and insulitic type 2 diabetic donors (*n* = 9, squares). Error bars show SEMs

When stimulated with high glucose in a dynamic perifusion assay, isolated islets from type 2 diabetic donors secreted less insulin, although with preserved dynamics, compared with age-, BMI- and sex-matched non-diabetic controls (*n* = 38). No differences regarding the dynamics and amounts of insulin release were found between insulitic (*n* = 9) and non-insulitic (*n* = 27) donors (Fig. 5).

### Type 2 diabetic donors with and without insulitis did not have different clinical characteristics

**Fig. 6:**
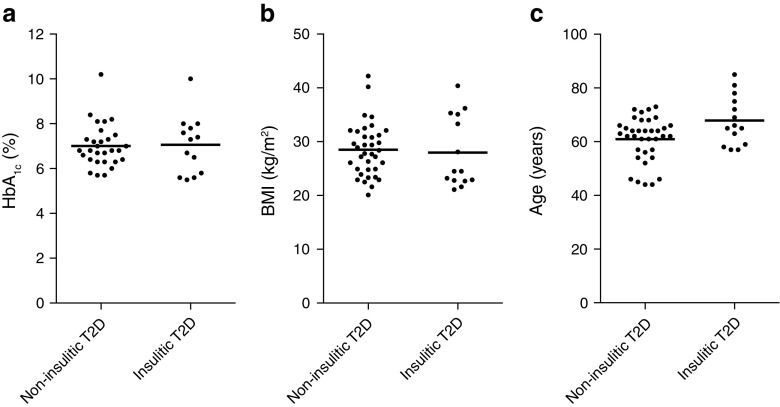
Clinical characteristics of non-insulitic and insulitic type 2 diabetic donors. Non-insulitic and insulitic type 2 diabetic donors were compared for HbA_1c_, BMI and age. Each black dot represents the value from one donor. The horizontal bar shows the mean value; *p* < 0.05 was considered statistically significant. To convert values for HbA_1c_ in DCCT % into mmol/mol, subtract 2.15 and multiply by 10.929. T2D, type 2 diabetes

There were no significant differences regarding BMI or HbA_1c_ between the non-insulitic and insulitic type 2 diabetic donors (Fig. 6a, b). Age was higher in the group of insulitic type 2 diabetic donors compared with non-insulitic donors (Fig. 6c). One type 2 diabetic donor had autoantibodies against GAD65, two donors had islet cell antibodies (ICA) and antibodies against GAD65 and one donor had ICA and antibodies against GAD65 and zinc transporter 8 (ZNT8). None of these autoantibody-positive donors fulfilled the criteria for insulitis.

## Discussion

The current definition of insulitis cannot be used to distinguish pancreases retrieved from individuals with type 1 diabetes from those with type 2 diabetes, as in this study insulitis was found in 31% and 28% of individuals, respectively. The biopsies analysed in this study were of high quality as the pancreases were procured from heart-beating organ donors. A limitation of our study is that the medical records of the donors were not made available in order to protect the integrity of the deceased person. However, most individuals had been diagnosed with type 2 diabetes for several years. In a meta-analysis, insulitis defined by the less stringent criteria of ≥ 5 CD45^+^ cells in ≥ 3 islets was present in 56% of individuals with type 1 diabetes examined within the first month of diagnosis and in only four of 132 individuals (3%) examined > 1 year after diagnosis [2].

The consensus definition of insulitis was adopted at the nPOD meeting in February 2013 and published in November 2013 in an effort to enhance understanding of the pathogenesis of type 1 diabetes and to allow comparisons between histopathological studies [1]. CD45 was chosen as a marker for immune cells because the insulitic infiltrates in type 1 diabetes include T cells (predominantly CD8^+^ but also CD4^+^ cells), B cells, macrophages and granulocytes [1, 3, 5]. This definition was designed to distinguish pancreases from individuals with type 1 diabetes from pancreases with background inflammatory infiltrates, as observed in non-diabetic individuals [1]. Since then, few publications have adopted this more stringent definition of insulitis. The findings presented in this study call for a revision of the definition in order to allow pancreases from individuals with type 1 diabetes to be distinguished from those with type 2 diabetes. Furthermore, in a recent study of non-diabetic autoantibody-negative donors, four of eight individuals fulfilled the criteria for insulitis [12], further reinforcing the need to revise the current definition of insulitis.

The highest number of CD3^+^ cells within or adjacent to an islet from an individual with type 2 diabetes in the present study was 18 and we found multiple islets containing 6–10 CD3^+^ cells. We therefore suggest that an insulitic islet should be redefined so that a lesion can be established when ≥ 15 CD3^+^ cells are present in one section, within the islet parenchyma or in the islet–exocrine interface. The disease process in type 1 diabetes is heterogeneously distributed within the pancreas [13]. Therefore, the presence of insulitis should preferentially be examined in multiple lobes of the pancreas. However, the limited availability of well-preserved human pancreases means this may not always be possible. The human pancreas contains several millions of islets of hugely varying size; small islets composed of only a few endocrine cells dominate and islets with a diameter of more than 250 μm are only rarely found [18–20]. Obviously, the size of an islet will influence the number of immune cells present per islet. Based on these considerations, specifying the number of islets in the definition of insulitis (i.e. ≥ 3 islets) should, if possible, be avoided. Instead, the proportion of islets with infiltrating CD3^+^ cells provides a better diagnostic criterion. In addition, the variation in islet size suggests that at least 50, but preferably 100, islets should be examined. In an effort to adhere to the consensus definition, it is proposed that if < 100 islets are available for evaluation, ≥ 3 islets should contain ≥ 15 CD3^+^ cells. However, if ≥100 islets are available for evaluation, ≥ 3% should contain ≥ 15 CD3^+^ cells (see text box: proposed definitions of insulitis). Using this definition for type 1 diabetes, both of the acute-onset type 1 diabetic donors included in this study and six out of six individuals with recent-onset type 1 diabetes examined within the Diabetes Virus Detection (DiViD) study fulfil the criteria [13], i.e. 11% of all examined islets in the DiViD study had ≥ 15 CD3^+^ cells. Furthermore, the proposed definition would adequately distinguish between individuals with type 1 diabetes and type 2 diabetes, as none of the 50 type 2 diabetic donors included in this study fulfilled these criteria. An adjusted definition of insulitis may also be applied to type 2 diabetes (see text box). The proposed definition for insulitis in type 2 diabetes was fulfilled by 28% of individuals with type 2 diabetes. This definition is, however, unable to discriminate between type 2 diabetes and type 1 diabetes. Therefore, based on the findings presented here, it is proposed that the discrimination of type 1 diabetes and type 2 diabetes should be based on the presence of insulin-deficient islets.

The recruitment of immune cells in type 2 diabetic pancreases has been proposed to be caused by beta cell stress [8]. However, the CD45^+^ cells found within the pancreases in this study were not preferentially located to the islets, but were seemingly randomly distributed throughout the entire gland. An increased presence of immune cells in the exocrine pancreas has been reported previously in both type 2 and type 1 diabetes [9, 10]. Accumulations of CD45^+^ cells were frequently found in areas of fibrosis, in which islets were densely surrounded by clusters of immune cells. Fibrosis constitutes the end stage of inflammation and it is likely that cellular inflammation maintained several years after diagnosis contributes to the loss of exocrine as well as endocrine parenchyma in individuals with type 2 diabetes [21]. Inflammation mediated by CD45^+^ cells, mainly CD68^+^ macrophages, may also directly affect islet function and survival, arguing for a direct role of these cells in the pathogenesis of type 2 diabetes. Supporting this notion, laser capture-microdissected islets from type 2 diabetic pancreases show cytokine and chemokine patterns that suggest a sustained mild inflammation affecting the islets [22, 23] and possibly contributing to islet dysfunction. This notion was not, however, supported by the functional characterisation of isolated islets in the present study. Even though we found a pronounced decrease in glucose-stimulated insulin release in islets isolated from pancreases obtained from individuals with type 2 diabetes when compared with islets from non-diabetic individuals, no difference in function could be observed between islets from type 2 diabetes donors with and without insulitis. Similarly, insulitis did not correlate with the level of HbA_1c_. These functional observations should, however, be interpreted with caution—technical limitations mean we do not have information on insulitis in the actual islets examined in vitro, and HbA_1c_ critically depends on the intensity of diabetes treatment.

No correlation could be found between insulitis and the presence of islet autoantibodies in the type 2 diabetic donors. This should, however, be interpreted with caution as only 8% of the individuals with type 2 diabetes in our study had one or more autoantibodies. Although markedly higher than in non-diabetic individuals, the frequency of autoantibodies in children or adolescents diagnosed with type 2 diabetes has in larger studies been reported to be as high as 30% [24]. Lack of insulitis in individuals with autoantibodies has been reported previously [25], and suggests that autoantibodies may appear in response to beta cell damage without being accompanied by a cellular immune response infiltrating the islets.

In addition to insulitis, other morphological characteristics of type 1 diabetes are also present in the pancreases of individuals with type 2 diabetes, for example HLA class I hyperstaining. However, the frequent finding of islets devoid of beta cells in pancreases obtained from individuals with recent-onset as well as long-standing type 1 diabetes is not found in individuals with type 2 diabetes. Thus, the appearance of insulin-deficient islets constitutes the main morphological criterion able to distinguish type 1 diabetic pancreases from type 2 diabetic pancreases, even though the loss of beta cells in type 1 diabetes varies greatly, especially at the onset of disease [13]. These observations indicate that leucocyte infiltration of islets has different immunopathological roles in type 1 diabetes and type 2 diabetes, a notion also supported by the higher proportion of T cells in the insulitic lesions in type 1 diabetes [2].

Insulitis is considered one of the major morphological hallmarks of type 1 diabetes. The findings presented show that the current consensus definition of insulitis [1] is not sufficient to separate the ongoing inflammation in the pancreas in individuals with type 1 diabetes from that occurring in those with type 2 diabetes. A modification of the definition of insulitis is therefore warranted. Based on our findings in a large cohort of type 2 diabetic donors, we propose that the diagnosis should be made when ≥ 15 CD3^+^ cells, not CD45^+^ cells, are found in ≥ 3 islets.

## Electronic supplementary material

Below is the link to the electronic supplementary material.

ESM Table 1 (PDF 61 kb)

## Abbreviations

DiViD: Diabetes Virus Detection
ICA: Islet cell autoantibodies
MPO: Myeloperoxidase
nPOD: Network for Pancreatic Organ Donors with Diabetes
