# Supplementary material for: Insulitis in human diabetes: a histological evaluation of donor pancreases
Source: Diabetologia. 2016 Oct 28;60(2):346–53. doi: 10.1007/s00125-016-4140-z (PMC6518093; doi:10.1007/s00125-016-4140-z)
Supplement: Supplementary file 1 — (PDF 61 kb) [file 125_2016_4140_MOESM1_ESM.pdf]

ESM Table 1. Information about primary antibodies

Primary Antibody	Manufacturer	Clone/article number	Antigen retrieval	Antibody dilution/concentration	Incubation time
Synaptophysin	Dako	DAK-SYNAP	pH 9	1/100	30
CD45	Dako	2B11+PD7/26	pH 9	1/75	30
CD68	Dako	KP1	pH 9	1/100	30
MPO	Dako	A0398	pH 9	1/1 200	30
CD3	Dako	A0452	pH 9	1/100	30
CD20	Dako	L26	pH 9	1/600	30
INS	Dako	A0564	pH 9	1/200	30
GLU	LSBIO	LS C312053	pH 6	1/400	30
HLA Class I ABC	Dako	EMR8-5	pH 6	1/1 500	60
Caspase 3	Abcam	Ab2302	pH 9	10 µg/ml	60
